# *Opt*ineurin Functions for *Opti*mal Immunity

**DOI:** 10.3389/fimmu.2018.00769

**Published:** 2018-04-10

**Authors:** Karolina Slowicka, Geert van Loo

**Affiliations:** ^1^Unit of Cellular and Molecular Pathophysiology, VIB Center for Inflammation Research, Ghent, Belgium; ^2^Department of Biomedical Molecular Biology, Ghent University, Ghent, Belgium

**Keywords:** optineurin, inflammation, immunity, mitophagy/autophagy, nuclear factor-κB, ubiquitin, TANK-binding kinase 1

## Abstract

Optineurin (OPTN) was identified 20 years ago in a yeast-two-hybrid screen with a viral protein known to inhibit the cytolytic effects of tumor necrosis factor. Since then, OPTN has been identified as a ubiquitin-binding protein involved in many signaling pathways and cellular processes, and mutations in the *OPTN* gene have been associated with glaucoma, Paget’s disease of bone and neurodegenerative pathologies. Its role in autophagy, however, has attracted most attention in recent years and may explain (some of) the mechanisms behind the disease-associated mutations of OPTN. In this brief review, we focus on the role of OPTN in inflammation and immunity and describe how this may translate to its involvement in human disease.

## Introduction

Optineurin (OPTN) was first identified as a binding partner of an adenoviral E3 14.7 kDa protein and named “FIP-2” (for E3-14.7K-interacting protein) but after renamed to “optineurin” (for *opti*c *neur*opathy *in*ducing) since mutations in the *OPTN* gene had been identified in patients with primary open-angle glaucoma (1, 2). Later on, OPTN mutations were also identified in other human pathologies including Paget disease of bone, amyotrophic lateral sclerosis (ALS) and frontotemporal dementia (FTD) (3–5), explaining the growing interest of the scientific community for this gene.

**Figure 1:**
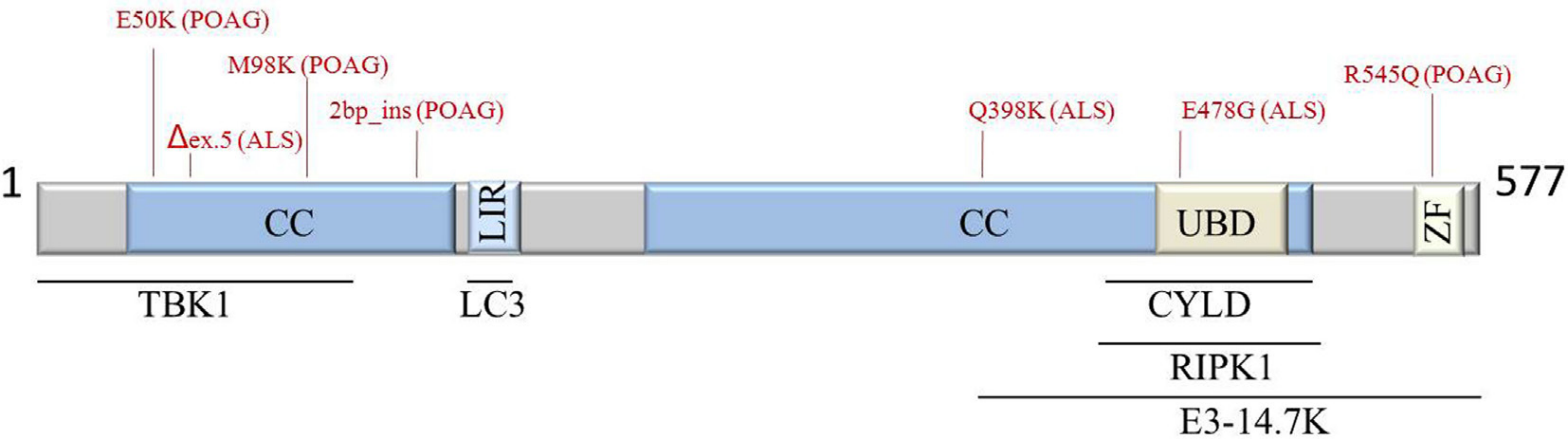
Schematic representation of OPTN protein structure, interaction partners, and most common POAG- and ALS-associated mutations (indicated in red). Abbreviations: ALS, amyotrophic lateral sclerosis; CC, coiled coil; CYLD, cylindromatosis; E3-14.7K, early region 3 14.7 kDa protein; LIR, LC3-interacting region; POAG, primary open-angle glaucoma; RIPK1, receptor-interacting protein kinase 1; TBK1, TANK-binding kinase 1; UBD; ubiquitin-binding domain; ZF, zinc finger; OPTN, optineurin.

Optineurin has been characterized as a multifunctional protein regulating multiple cellular processes such as vesicular trafficking, cell division, inflammatory and antiviral signaling, anti-bacterial responses, and autophagy. OPTN can bind multiple partners; hence, disease-causing mutations may alter these interactions disturbing normal signaling (Figure 1). However, many questions remain, and more evidence is needed to clarify its multiple functions and contribution to disease.

## OPTN and Nuclear Factor-κB (NF-κB) Signaling

**Figure 2:**
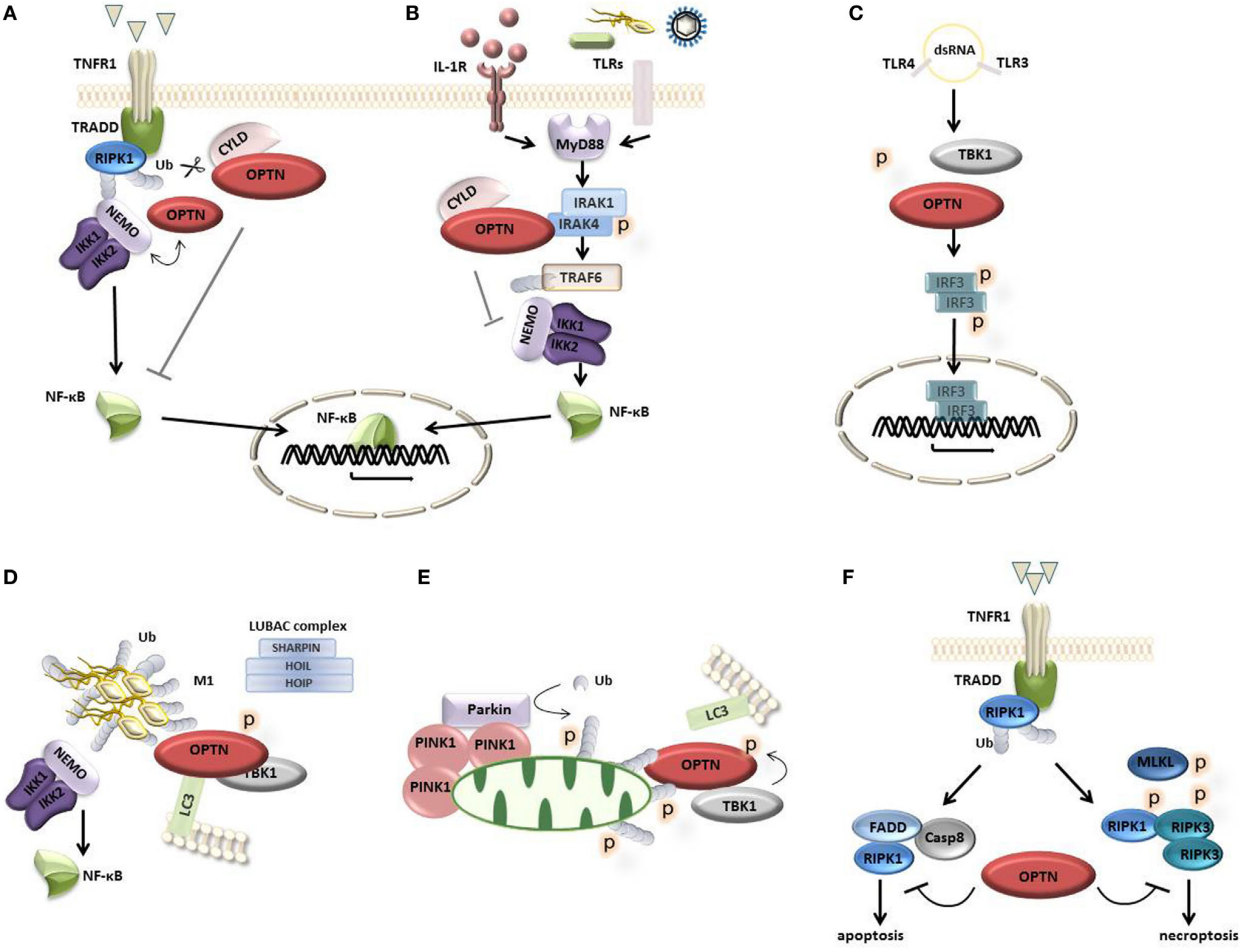
Optineurin (OPTN) in cellular signaling. **(A)** Upon activation of tumor necrosis factor (TNF) receptor 1, OPTN is recruited to the receptor complex where it competes with nuclear factor-κB (NF-κB) essential modulator (NEMO) for ubiquitinated RIPK1. OPTN can also recruit the deubiquitinating enzyme cylindromatosis (CYLD), which cleaves off ubiquitin chains from proteins such as NEMO and RIPK1, blocking downstream NF-κB signaling. **(B)** Upon activation of IL-1 and TLR receptors with their respective ligands, the adaptor protein MyD88 is recruited to the receptor complex where it engages the kinase interleukin-1 (IL-1) receptor-associated kinase 1 (IRAK-1) and the E3 ligase TRAF6, inducing TRAF6 auto-ubiquitination and downstream NF-κB signaling. OPTN can bind IRAK-1 through its ubiquitin-binding domain preventing TRAF6 ubiquitination and suppressing downstream NF-κB signaling. This process may involve the recruitment of CYLD. **(C)** Bacterial LPS and viral double-stranded RNA (dsRNA) induce the activation of TANK-binding kinase 1 (TBK1), which binds and phosphorylates OPTN, leading to the phosphorylation, dimerization, and nuclear translocation of IFN regulatory factor 3 (IRF3), mediating the transcription of interferon type I response genes. **(D)** OPTN acts as an autophagy receptor that associates with ubiquitin-coated cytosolic bacteria and recruits TBK1 to phosphorylate OPTN, enhancing its LC3 binding affinity, directing autophagosomal degradation. The LUBAC complex (consisting of Sharpin, HOIL-1, and HOIP) mediates the linear ubiquitination at the bacterial surface inducing the recruitment of NEMO and OPTN to activate NF-κB signaling and autophagy. **(E)** Damaged mitochondria are removed through mitophagy, a process involving the kinase PINK1 and the E3 ubiquitin ligase Parkin, which conjugates ubiquitin to mitochondrial outer membrane proteins, recruiting OPTN. This process involves TBK1-dependent OPTN phosphorylation promoting the autophagosomal degradation of mitochondria. **(F)** OPTN can bind caspase 8, limiting complex II formation and apoptosis. Alternatively, OPTN can regulate the turnover of ubiquitinated RIPK1 protecting from necroptosis.

Inflammatory signaling pathways, and especially NF-κB signaling, are heavily controlled by ubiquitination, a posttranslational modification of proteins. Polyubiquitin chain formation through lysine 48 (K48) of ubiquitin directs proteasomal degradation of the modified protein. By contrast, K63 or linear (M1) ubiquitination normally do not lead to degradation of the protein but mediate the binding of other proteins that contain specific ubiquitin-binding domains (UBDs), driving downstream signaling (6). OPTN’s sequence shows striking homology to NF-κB essential modulator (NEMO), the core regulatory element of the inhibitor of NF-κB kinase (IKK) complex essential for NF-κB activation (7). Both OPTN and NEMO have a similar UBD which facilitates binding to M1 and K63-linked ubiquitin chains, but not to the K48-linked ones (8). Despite their sequence homology, OPTN cannot substitute NEMO in the IKK complex (9). Initially, *in vitro* studies identified OPTN as a negative regulator of NF-κB signaling in response to tumor necrosis factor (TNF) by competing with NEMO for binding to ubiquitinated receptor-interacting protein kinase 1 (RIPK1), dampening downstream inflammatory signaling (7, 10) (Figure 2A). Recent structural studies showed that linear ubiquitin binding by the OPTN UBD is critical for NF-κB suppression (11). OPTN was also shown to interact with cylindromatosis (CYLD), a deubiquitinating enzyme that cleaves linear and K63-linked ubiquitin chains from proteins such as NEMO and RIPK1, to block downstream NF-κB signaling (12). OPTN has also been identified as a binding partner of interleukin-1 (IL-1) receptor-associated kinase 1 (IRAK-1), where it suppresses NF-κB activation in response to IL-1β and toll-like receptor stimulation by preventing the poly-ubiquitination of TRAF6 (13) (Figure 2B). A mutant version of OPTN that fails to recruit CYLD to inhibit NF-κB activation in response to TNF is also unable to inhibit IRAK-1-induced NF-κB signaling (12, 13). Finally, OPTN was recently shown to suppress T cell receptor-induced NF-κB activation and TNF production, in a manner dependent on ubiquitin-binding (14).

In contrast to these *in vitro* studies, *in vivo* studies do not confirm a role for OPTN in NF-κB signaling. OPTN[D477N] knock-in mice expressing a point mutation abolishing its polyubiquitin-binding activity, as well as mice lacking either the entire C-terminal UBD or the N-terminal TBK1-binding domain, show normal NF-κB responses (8, 15, 16). Also, OPTN deficiency does not affect TNF nor TLR-induced NF-κB activation, arguing against a role for OPTN in the regulation of NF-κB signaling *in vivo* (17).

## OPTN and Interferon (IFN) Signaling

The production of type I IFNs is an essential initial step in the host defense against infections (18). Bacterial LPS and viral double-stranded RNA (dsRNA) induce immune responses through the activation of TBK1, the phosphorylation of IFN regulatory factor 3 (IRF3), and the production of type I IFNs. OPTN can bind to TBK1, but not to the related kinase IKKε (8, 19). Bone marrow derived macrophages (BMDMs) from OPTN-deficient mice (17) or from OPTN mutant mice lacking the UBD or the TBK1-interacting region (15, 16) show diminished phosphorylation of TBK1 and IRF3 and as a result secrete lower levels of IFN-β in response to LPS or the dsRNA mimetic poly(I;C) (Figure 2C). Also BMDMs from ubiquitin-binding-defective OPTN[D477N] mutant mice show reduced TBK1 activity, IRF3 phosphorylation and production of IFN-β in response to LPS or poly(I;C) (8). Recent work from Bakshi and colleagues indeed shows that the interaction between ubiquitin chains and OPTN is required for robust phosphorylation and activation of the OPTN-TBK1 complex, triggering IRF3 activation and IFN-β production (20).

## OPTN and Autophagy

With the identification of OPTN as an autophagy receptor, a new era of research on OPTN started. Autophagy is a lysosomal degradation pathway important for the removal of protein aggregates, intracellular bacteria and damaged cellular organelles (21). In 2011, the group of Ivan Dikic demonstrated that OPTN can bind to the autophagy protein LC3 *via* an LC3-interacting (LIR) motif, and with ubiquitin *via* its UBD. Upon infection of cells with *Salmonella*, OPTN associates with ubiquitin-coated bacteria and recruits TBK1 that phosphorylates OPTN, enhancing its LC3 binding affinity, through which it promotes the autophagic clearance of bacteria (22, 23). Also *in vivo*, OPTN was shown to control *Salmonella* infection (17, 22, 23). Upon infection, invading *Salmonella* bacteria become decorated with ubiquitin chains that serve as a platform to trigger various signaling cascades (24). This coat of ubiquitin around the bacteria is not uniform but contains distinct patterns of both linear and K63-linked chains eliciting different downstream signaling pathways (24, 25). Linear ubiquitination at the bacterial surface induces the recruitment of OPTN and NEMO, activating selective autophagy and inducing IKK activation and NF-κB-dependent inflammatory signaling, respectively, restricting bacterial proliferation (24, 25). The origin of M1-linked polyubiquitin in the ubiquitin coat on the bacterial surface is most probably caused by the localized recruitment of the M1-specific E3 ubiquitin ligase complex LUBAC (25) (Figure 2D). Besides OPTN, also other autophagy receptors, such as p62 and NDP52, are recruited to autophagosomal membranes through their LIR motifs. However, the recruitment of p62 and NDP52 occurs independently of LUBAC (25), demonstrating that, although there are multiple autophagy receptors, their functions are not completely redundant. Instead, they depend on their specific interacting partners and the downstream signaling cascades they activate.

Besides its role in the autophagy-mediated elimination of intracellular pathogens (xenophagy), OPTN also controls the selective autophagy of damaged mitochondria which also become conjugated with ubiquitins. This process of mitophagy depends on the kinase PINK1 and the E3 ligase Parkin, which, upon activation, ubiquitinates mitochondrial outer membrane proteins to recruit autophagy receptors. Mitophagy also involves TBK1 activation, leading to its translocation to mitochondria where it phosphorylates OPTN thereby expanding the binding capacity of OPTN to diverse ubiquitin chains, directing ubiquitin-loaded mitochondria into autophagosomes (Figure 2E) (23, 26, 27).

## OPTN and Disease-Associated Mutations

Considerable interest in OPTN came from the identification of mutations in the *OPTN* gene in patients with degenerative diseases such as glaucoma, ALS, and FTD. Although many hypo-theses explaining the consequences of these mutations have been suggested, clear mechanisms of pathogenesis caused by OPTN mutations are still not clear.

Several OPTN mutations have been identified in patients with ALS and FTD (4, 28–31). Also mutations in TBK1 have been linked with these diseases (5, 32, 33), suggesting a common pathway defect in these pathologies. The ALS-associated TBK1 E696K mutation specifically abolishes its binding to OPTN and disrupts OPTN/TBK1 complex formation (23, 34), this in contrast to the glaucoma-associated OPTN E50K mutation, shown to cause death of retinal ganglion cells *in vitro* and in transgenic mice (35), which enhances the interaction between OPTN and TBK1, affecting the oligomeric state of OPTN (34). However, many ALS-associated OPTN mutations map to the C-terminal part of the protein, and not to the N-terminal TBK1-binding region, making it unlikely that these mutations will affect the interaction between OPTN and TBK1. These mutations may, however, disturb the ubiquitin-binding function of OPTN and may hint to a defect in the process of selective autophagy (34) or to a defect in OPTN’s ability to suppress NF-κB activation and apoptosis *via* linear ubiquitin binding (11). A recent study suggests that OPTN protects from neurodegeneration and ALS by suppressing RIPK1-dependent signaling and necroptosis, a form of regulated necrotic cell death (Figure 2F) (36). Indeed, OPTN-deficient mice develop progressive demyelination and axonal degeneration, reminiscent of ALS, due to CNS cell necroptosis and neuroinflammation, a phenotype which could be rescued by inhibiting RIPK1 kinase activity preventing necroptosis in OPTN-deficient mice (36). OPTN has also been shown to protect motor neurons from TNF-induced apoptosis, through association with caspase 8 at its dead effector domains to prevent the recruitment of FADD and downstream caspase activation (11). In agreement, cleaved caspase 3 was detected in motor neurons from OPTN-associated ALS patients, suggesting enhanced apoptosis in the absence of OPTN (11). Besides these signs of motor neuron apoptosis, intracytoplasmic inclusions in brain samples from patients with OPTN mutations often stain positive for linear ubiquitin and activated NF-κB (11).

Genetic variants of *OPTN* leading to lower OPTN expression have also been identified in patients with Paget’s bone disease (3, 37). OPTN was shown to act as a negative regulator of osteoclast differentiation, and mice with a loss-of-function mutation in the UBD of OPTN have increased osteoclast activity and bone turnover (38).

Finally, OPTN has been associated with Crohn’s disease (CD), and diminished expression of OPTN was observed in approximately 10% of CD patients (39, 40). Patient-derived macrophages show decreased pro-inflammatory cytokine secretion, suggesting an effect on inflammatory responses and bacterial clearance, as is observed in CD (39, 40). In agreement, loss of OPTN in mice was shown to impair cytokine production and neutrophil recruitment in a bacteria-dependent model of colitis (40, 41). However, contrary to the bacteria-driven colitis, OPTN knockout mice respond normally to the model of dextran sodium sulfate-induced colitis (17, 41). This difference in response between both colitis models might suggest a specific role for OPTN in protection from bacterial infection and infection-associated IBD. Recently, OPTN has been implicated in CD through its interaction with the endoplasmic reticulum (ER) stress sensor IRE1α, *via* which it was suggested to participate in the removal of ER membranes in conditions of prolonged ER stress (42). Defective autophagy and ER stress in intestinal epithelial cells induce IRE1α aggregation, triggering intestinal inflammation. However, these IRE1α aggregates can be recruited to autophagosomes *via* OPTN, followed by IRE1α degradation (42). Hence, OPTN-dependent selective autophagy (ERphagy) may act as a mechanism to protect from prolonged ER stress and intestinal inflammation. Alternatively, since OPTN has previously been shown to localize to protein aggregates (43), OPTN could recognize the misfolded proteins and mediate their clearance independently of IRE1α.

## Concluding Remarks

Optineurin has been implicated in many signaling pathways and cellular processes. Overall, three major protective mechanisms can be considered: regulation of selective autophagy, regulation of inflammatory signaling, and protection from cell death. Mutations in OPTN, interfering with these protective activities, may eventually lead to disease. However, despite our knowledge on the role of OPTN in these cellular processes, we still know very little concerning the molecular mechanisms behind the disease-associated OPTN mutations. Most of these mutations have only been studied upon overexpression in cell culture models and have never been validated *in vivo*. Since OPTN knockout mice nor OPTN mutant mice defective in ubiquitin binding (15–17) develop spontaneous disease, OPTN mutations most probably induce a gain-of-function, rather than a mere loss of its normal function. Hence, the development of OPTN knock-in models expressing specific disease-associated mutations will be crucially important to clarify the importance of these mutations for disease development and will help to better understand the biological functions of OPTN.

## Author Contributions

All authors listed have made a substantial, direct, and intellectual contribution to the work and approved it for publication.

## Conflict of Interest Statement

The authors declare that the research was conducted in the absence of any commercial or financial relationships that could be construed as a potential conflict of interest.
